# LncRNA FAM30A Suppresses Proliferation and Metastasis of Colorectal Carcinoma by Blocking the JAK–STAT Signalling

**DOI:** 10.1111/jcmm.70421

**Published:** 2025-02-19

**Authors:** Jin Liu, Shuangyin Han, Yuanbo Cui, Qiuyan Zhao, Yunfei Wang, Tian Li, Xiuling Li

**Affiliations:** ^1^ Department of Gastroenterology Henan Provincial People's Hospital Zhengzhou China; ^2^ Trauma and Metabolic Institute Zhengzhou Central Hospital Affiliated to Zhengzhou University Zhengzhou China; ^3^ Tianjin Key Laboratory of Acute Abdomen Disease‐Associated Organ Injury and ITCWM Repair, Institute of Integrative Medicine of Acute Abdominal Diseases Tianjin Nankai Hospital, Tianjin Medical University Tianjin China

**Keywords:** colorectal carcinoma, FAM30A, JAK–STAT signalling, lncRNA

## Abstract

Colorectal carcinoma (CRC) poses a serious risk to global human health. Long non‐coding RNAs (LncRNAs) play an important role in the pathogenesis of CRC. There is a scarcity of data about a newly identified lncRNA, FAM30A. Our major objective is to investigate the role of FAM30A in the process of CRC. Gene expression data and correlated clinical information were retrieved and downloaded from public databases to identify differentially expressed genes linked to CRC. The expression of FAM30A was identified in clinical samples and CRC cell lines using via Quantitative Real‐time Polymerase Chain Reaction (qPCR) assay also. The survival significance of FAM30A was determined via R package “survival.” Kyoto Encyclopedia of Genes and Genomes (KEGG) enrichment analysis was performed to identify FAM30A‐related signalling pathway. The levels of proteins expression were determined by western blot assay. The effect of FAM30A on CRC cell biological behaviours was evaluated by cell function experiments. FAM30A was identified down‐regulated in CRC based on the data from public database. FAM30A had lower expression in CRC clinical samples and cell lines. Low FAM30A expression was positively related to a poor prognosis in CRC patients. After FAM30A was overexpressed, the proliferation, invasion, and migration abilities of CRC cells were decreased, and the rate of CRC cell apoptosis increased. Furthermore, overexpression of FAM30A could block JAK–STAT signalling. FAM30A suppresses proliferative, invasive, and migratory abilities of CRC through blocking JAK–STAT signalling. Thus, it can be a novel biomarker of CRC prognosis.

**Abbreviations**
CRC: colorectal carcinoma
FISH: fluorescence in situ hybridisation
GEO: Gene Expression Omnibus
HR: hazard ratio
ncRNAs: non‐coding RNAs
TCGA: The Cancer Genome Atlas

## 1 Introduction

Neoplasms, commonly known as tumours, continue to be the leading cause of mortality around the globe. Despite advancements in medical science and technology, various forms of cancer—whether malignant or benign—pose significant health risks and account for a substantial number of deaths each year [1, 2, 3, 4]. Among which, colorectal carcinoma (CRC) is one of the top three most common cancers diagnosed, as it accounts for about 10% of all cancer cases and cancer‐related deaths worldwide each year [4, 5, 6, 7, 8, 9]. The worldwide occurrence of CRC is projected to increase to 25 million over the next 15 years [10], and the exact reasons for this increase are not fully understood [11]. Therefore, it is urgent to find novel therapeutic targets that may facilitate to understand the pathogenesis of CRC and reduce the huge burden of colorectal cancer on society and families. CRC is complex at the molecular level. Incomplete genome, damaged DNA mismatch repair, and abnormalities in histone modification or DNA methylation may induce carcinogenesis in normal epithelial cells epigenetically [12, 13]. Methylation, RNA splicing defects, and protein synthesis abnormalities also alter the normal expression of colorectal cancer proto‐oncogenes or tumour suppressor genes during post‐transcriptional modification [14, 15]. Previous studies have shown that several cell signalling pathways play an important role in the progression of cancers include CRC, such as Hippo, JAK/STAT, NOTCH, SHH/GLI, TGF/SMAD, and Wnt/beta‐catenin [16, 17]. CRC exhibits significant biological heterogeneity, which means that tumours can vary widely in their genetic and molecular characteristics. This heterogeneity can lead to differences in treatment responses among patients, making it difficult to find universally effective therapies [18, 19]. Therefore, research studies focusing on molecular function can provide the precision treatment of CRC with more guidance.

While 93% of human genomic DNA can be transcribed into RNA, only about 2% of them can encode proteins, and most of them are non‐coding RNAs (ncRNAs). ncRNAs are categorised by length into small ncRNAs (< 50 nt, including miRNA, siRNA, and piRNA) and long non‐coding RNAs (lncRNAs; > 200 nt, lincRNA and circRNA) [20, 21]. LncRNAs were originally considered to be “noise” in the genome because of lacking open reading and coding functions [22, 23, 24, 25]. The biological roles of lncRNAs are constantly being discovered with deeper research studies. LncRNAs possess a preserved secondary structure that can interact with DNA, RNA, and proteins, thereby influencing the transcription and translation of functional genes. At the transcriptional level, gene expression can be regulated by lncRNA‐mediated cis‐regulation, DNA–RNA‐protein complex, competition binding with transcription factors and complementary antisense strands. At the post‐transcriptional level, lncRNAs can regulate the modification and degradation of mRNA, and regulate protein synthesis, transport, and ubiquitination [26, 27]. LncRNA can also regulate gene expression by regulating DNA methylation modification, histone acetylation, or methylation modification and chromatin topology, epigenetically. With the comprehensive application of sequencing technology and bioinformatics analysis combined with large clinical samples, the functional mechanism of cancer‐related lncRNAs has been deeply studied, and these lncRNAs could therefore be applied as potential therapeutic targets, diagnostic and prognostic molecular markers. LncRNAs have gradually become a hot spot of current medical research. Lots of lncRNAs are said to play vital role in CRC. For instance, lncRNA MALAT1 can effectively suppress the spread of CRC cells [28] and influence the migration and invasion process of CRC cells by regulating AKAP9 [29, 30]. In vitro experiments have shown that up‐regulation of HOTAIR, the first identified trans‐transcriptional regulated lncRNA, can increase the malignant transformation of CRC cells, and down‐regulation of HOTAIR can inhibit malignant phenotypes such as metastasis and invasion to a certain extent [31].

The research of lncRNA combine cell signalling pathway is a promising approach to explore the mechanism of inhibiting CRC [32]. This research concentrated on the function of FAM30A, a newly found lncRNA in CRC. Through an extensive examination of Gene Expression Omnibus (GEO) microarray data and TCGA sequencing data, we pinpointed the differentially expressed lncRNA FAM30A as the target of our research. Subsequently, we confirmed FAM30A expression in clinical samples and multiple CRC cell lines, and investigated its biological impact on CRC cells along with the associated molecular mechanisms.

## 2 Methods

### 2.1 Data Source and Preprocessing

Gene expression data from four datasets, GSE9348 [33], GSE32323 [34], GSE8671 [35], and GSE39582 [36], obtained from the GEO were used to assess the expression of FAM30A in CRC. Detailed information of each dataset can be found in Table S1. GSE9348 (Singapore cohort), GSE32323 (Japanese cohort), GSE8761 (Switzerland cohort), and GSE39582 (French cohort) represented CRC patients in different countries and regions, and the aim was to explore common differentially expressed genes (DEGs) through these four CRC datasets. RNA sequencing data and related clinical details for 165 CRC samples were obtained from The Cancer Genome Atlas (TCGA) project through the Genome Data Commons Data Portal (https://portal.gdc.cancer.gov). RNA sequencing data, counts data were converted to counts per million reads (CPM) based on the qCML method using edgeR package of R language [37]. Then, CPM was converted to log2 (CPM + 1) for further analysis. Negative binomial distributions combined with Fisher's exact was used to calculate *p*‐values.

### 2.2 Survival Model Construction

Using the survival package [38] of R language, hazard ratio value of the cox risk regression model and the chi‐square test *p*‐value for the survival of the high and low groups based on TCGA dataset were calculated. According to the FAM30A expression median, CRC samples were categorised into two groups, one with elevated PAM30A expression and the other with reduced expression. A Kaplan–Meier survival analysis was conducted using pertinent clinical data accessed from TCGA, and finally the overall survival curve was drawn.

### 2.3 Estimation of Tumour Immune Microenvironment Scores

The tumour immune microenvironment scores of the samples accessed from TCGA were evaluated by ESTIMATE algorithm [39]. The scores included the tumour purity, the level of stromal cells, and the level of immune cell infiltration in tumour tissue. Subsequently, a correlational analysis was conducted to examine the relationship between gene expression and scores of tumour immune microenvironment.

### 2.4 KEGG Enrichment Analysis

DAVID [40], an online bioinformatics tool, was used to perform the KEGG enrichment analysis. Genes co‐expressed with FAM30A were inputted, with the entire human genome serving as the reference. The hypergeometric distribution test was employed to evaluate the disparity between the count of genes in a KEGG pathway and the count of genes in the background set that belong to the same pathway.

### 2.5 Co‐Expressed Genes Identification

GEPIA (http://gepia.cancer‐pku.cn/) [41], an online bioinformatics tool, created by Peking University for analysing RNA sequencing expression data from TCGA and GTEx projects. The similar genes detection module was employed to identify the co‐expressed genes of FAM30A in the TCGA COAD datasets. We selected genes with Pearson's correlation coefficient exceeding 0.50 as the co‐expressed genes of FAM30A for further analysis.

### 2.6 Patients and Clinical Specimens

Fresh specimens from 30 CRC patients (specific information about all patients was listed in Table S2) were obtained during surgery, and non‐tumour adjacent tissues (2 cm from the tumour tissue) were obtained as control. This recruitment started in April 2020 and concluded in July 2023, the clinical tissue samples collection and experiments were completed well before July 2023. Each patient had signed the informed consent documents. All aspects have been approved by the committee of Henan Provincial People's Hospital with the approval number of [2020]‐03. All patients did not receive chemotherapy, radiotherapy, or immunotherapy before surgery, and the influence of other serious diseases was excluded. The Paraffin sections of colon tissues were dewaxed, rehydrated, and stained in haematoxylin dye solution at 25°C for 5 min. The segments were cleaned using tap water. The washed sections were then immersed in 1% hydrochloric acid alcohol solution for several seconds and rinsed by tap water until they were back to blue colour. The segments were stained by eosin for 3–5 min and washed in tap water once again. Following dehydration, clearing, and sealing, the nuclei appeared as purple blue or blue, the cytoplasm showed pink, and red blood cells showed red.

### 2.7 Cell Culture

Human normal colonic epithelial cell lines (NCM460) and the CRC cell lines (Caco‐2, HCT116, LoVo, and SW480) were purchased from the Type Culture Collection of the Chinese Academy of Sciences (Shanghai, China). HCoEpiC cells were provided by the Henan International Joint Laboratory of Immunity and Targeted Therapy for Liver‐Intestinal Tumours, Xinxiang Medical University, Xinxiang, China. At the time of donation, the cells had just undergone their third passage. All the cell lines were sustained in RPMI‐1640 media (Gibco, Waltham, MA, USA) containing 10% foetal bovine serum (Gibco), 100 U/mL penicillin, and 100 mg/mL streptomycin (Invitrogen, Carlsbad, CA, USA) within a humidified incubator set to 37°C with 5% CO_2_.

### 2.8 Cell Transfection

FAM30A overexpression vector (pcDNA 3.1 FAM30A), which was used for inducing FAM30A overexpression and empty vector (pcDNA3.1) were got from GeneChem Company (Shanghai, China). Following the extraction of CRC cells from the incubator, they were subjected to trypsin digestion and subsequently counted. Cells were seeded into six‐well plates at a density of 5 × 10^3^ cells per well. Following 12 h of incubation, the cell density would reach 50%–70% of the total density of the culture dish, and then transfection was performed. Cells were transfected at a 50 nM concentration using Lipofectamine 2000 (Invitrogen; Thermo Fisher Scientific Inc.) according to the manufacturer's guidelines. After 48 h of transfection, the samples were collected for further analysis.

### 2.9 Real‐Time Quantitative PCR

Total RNA was isolated from cells using the RNeasy Mini Kit (QIAGEN, Hilden, Germany). Superscript IV Reverse Transcriptase (ThermoFisher, Waltham, MA, USA) was utilised to carry out reverse transcription PCR. SYBR green gel dye (Merck KGaA, Darmstadt, Germany) was used for DNA staining. Gene fold changes were determined by the $2^{-\Delta \Delta C_{t}}$ algorithm. U6 and GAPDH were used as reference genes to normalise $2^{-\Delta \Delta C_{t}}$‐based assessments. The specific primer sequences for FAM30A, U6, and GAPDH are shown in Table 1.

**TABLE 1:** qPCR primers for FAM30A, U6, and GAPDH.

	Forward	Reverse
FAM30A	CTGTGGCAAAGGCAAGTGAC	TTTCTTCCCTGTGCGGAGTC
U6	GCTTCGGCAGCACATATACTAAAAT	CGCTTCACGAATTTGCGTGTCAT
GAPDH	GGTGAAGGTCGGAGTCAACG	CAAAGTTGTCATGGATGHACC

### 2.10 Nuclear and Cytoplasmic Separation

Cultured CRC cells were collected and resuspended in cell lysate buffer (1% NP40, 5 nM EDTA, 0.5% sodium deoxycholate) for 5 min and then centrifugated at 4000 rpm for 1 min at 4°C. The supernatant and pellets were separately collected. Pellets were then washed with cell lysate buffer for 10 min at 4°C and followed by centrifuged for 5 min at the same temperature. Supernatant and pellets were further used for real‐time quantitative PCR.

### 2.11 Fluorescence In Situ Hybridisation

HCT116 and SW480 CRC cells were placed into 48‐well plates at a density of 1 × 10^4^ cells per well and were kept at 37°C. After 16 h of incubation, the cell density would reach 50%–70% of the total density of the culture dish, and then fluorescence in situ hybridisation (FISH) was performed. CRC cells were first washed with 1 × phosphate buffered saline (PBS, Sangon Biotech) twice, and then fixed for 15 min with 4% paraformaldehyde (Sangon Biotech). Removed 4% paraformaldehyde and washed cells with 1 × RNase free‐PBS for two times, each time lasted 5 min. Cells were treated and stabilised at ambient temperature with 0.1%Triton X‐100 (TritonX‐100, Sigma‐Aldrich). The sample was then treated to reduce signal of the background. Finally, FISH was performed and ImageJ software (Version 1.5.3) was used to analyse the results. The FAM30A probe sequence was as follows: 5′‐CATGGGGTATGGACTTAGGGTCTCA‐3′, and the probe was labelled with FITC. This assay was repeated for three times.

### 2.12 Cell Viability Assay

The Cell Counting Kit 8 (CCK‐8, Dojindo, Shanghai, China) was used to detect the cell viability. Transfected HCT116 and SW480 cell lines were seeded into 96‐well plates at a density of 5 × 10^3^ cells per well and incubated at 37°C for 0, 24, 48, 72, and 96 h. Subsequently, CCK‐8 solution (10 μL) was added into each well and incubated for 4 h at 37°C. Optical density value was determined at 450 nm with a microplate reader. To perform tumour sphere formation assay, CRC cells were cultured in six‐well ultra‐low attachment plate in serum‐free DMEM/F12 supplemented with B27, EGF (20 ng/mL), bFGF (20 ng/mL), and heparin (4 μg/mL). After 2 weeks, the spheres were photographed and counted.

### 2.13 EdU Assay

EdU assay with Cell‐Light EdU DNA Cell Proliferation Kit (RiboBio, Guangzhou, China) was used to detect Cell proliferation. Transfected cells were incubated at 37°C with 5% CO_2_ for 48 h, then EdU (50 mM) was added and incubated for an addition 2 h. The cells were then fixed with 4% paraformaldehyde and stained with Apollo Dye Solution. DAPI was used to stain nucleic acids in all cells. Fluorescence microscope was used to take images, and ImageJ (Version 1.5.3) software was used to calculate the cell proliferation rate.

### 2.14 Tumour Sphere Formation Assay

HCT‐116 and SW480 cells were collected and dissociated into single‐cell suspensions in serum‐free medium. Following precise cell counting, 200 cells per well were placed into 200 μL of the same medium onto a clear round bottom ultra‐low attachment microplate (Corning) with 10 wells dedicated for each cell line. The medium was refreshed every 48 h. Images from five randomly selected fields per group were captured using a fluorescence microscope (Leica, Wetzlar, Germany) after 7 days of incubation. The efficiency of sphere formation was calculated by dividing the number of formed spheres by the initial 200 cells seeded. The experiment was conducted three times, each with three biological replicates.

### 2.15 TUNEL Staining

A one‐step TUNEL apoptosis assay kit (Beyotime, Shanghai, China) was used to detect CRC cell apoptosis. Hanks' balanced salt solution (HBSS, Gibco, CA, USA) was replaced with normal medium for 6 h to reoxygenate the cells. Cells in six‐well plates were washed three times in PBS and fixed with 4% paraformaldehyde for 30 min at 25°C. Fixed cells were rinsed three times with PBS and cultured in 3% H_2_O_2_ methanol in the dark for 20 min. After washing three times with PBS, cells were cultured at 37°C for 1 h in a mixture of TdT and dUTP (1:9) in a TUNEL kit (Roche, Mannheim, Germany) at 37°C for 1 h, and then in converter‐POD at 37°C for 30 min. Subsequently, cells were cultured with DAPI for nuclear staining. TUNEL‐positive cells were observed under a light microscope. Each experiment was repeated three times independently.

### 2.16 Transwell Invasion Assay

SW480 and HCT116 cells were placed into 24‐well plates at a density of 5 × 10^4^ cells per well and incubated in a cell incubator for 20 h. Following transfection, the culture continued for an additional 24 h. Then, cells were digested and counted, and 1 × 10^5^ cells were seeded into each transwell chamber coated with Matrigel (1:8, 80 μL) and serum‐free DMEM medium (100 μL). Complete medium was added to the lower chamber of the transwell chamber, and after 24 h of culture, cells in the upper chamber were wiped with a cotton swab. Cells were fixed with 4% paraformaldehyde for 15 min and stained with crystal violet for 10 min. Under the microscope, five fields of view were randomly selected to be photographed and counted. The experiment was repeated three times, and the average was calculated.

### 2.17 Wound‐Healing Assay

SW480 and HCT116 cells were placed in six‐well plates at a density of 3 × 10^5^ cells per well and incubated for 20 h. On the back of the six‐well plate, a uniform horizontal line was drawn and cells were scratched with a pipette (10 μL) along a ruler perpendicular to the horizontal line on the back, and scraped cells were removed by rinsing with PBA. After transfection treatment, culture medium was added. Samples herein collected at 0 and 48 h, and the scratch‐healing area was calculated using the following formula. The experiment was repeated in triplicate, and the average value was calculated.

$$\text{Mobility}=\frac{\text{Scratch}-\text{healing area}}{\text{Scratch initiation area}}\times 100\%$$

### 2.18 Western Blot

Protein was extracted from colon tissue by RIPA lysis buffer (Beyotime Biotechnology). SDS‐PAGE was used to separate the protein samples (60 μg), and PVDF membranes (microwells) were used for transfer. The extracted protein was then incubated with the primary antibody (JAK1 antibody, 1:1000, cat. no. ab133666; p‐JAK1 antibody, 1:1000, cat. no. ab138005; STAT3 antibody, 1:1000, cat. no. ab68153; p‐STAT3 antibody, 1:1000, cat. no. ab267373; JAK2 antibody, 1: 1000, cat. no. ab170718; p‐JAK2 antibody, 1: 1000, cat. no. ab32101; STAT1 antibody, 1: 1000, cat. no. ab109461; p‐STAT1 antibody, 1: 1000, cat. no. ab307835; STAT5 antibody, 1: 1000, cat. no. ab16276; p‐STAT5 antibody, 1: 1000, cat. no. ab278764; Abcam, Cambridge, MA, USA) overnight at 4°C after blocking. The next day, Horseradish Peroxidase (HRP)‐conjugated secondary antibodies were used for the incubation. A fluorescence imager (Alpha) was used for visualisation and the expression levels of specific proteins were normalised to β‐actin levels.

### 2.19 Statistical Analysis

Microsoft excel was used to performed statistical analyses and visualisations, GraphPad Prism 8.0 or R software (version 4.1.0) except as otherwise noted. Clinical characteristics were expressed as mean ± standard deviation or *n* (%). The paired Student's *t* test was utilised to comparison of two paired group. Multiple groups were compared with one‐way ANOVA and Tukey's multiple comparisons test. The Benjamini–Hochberg method was used to control the false discovery rate. Adjusted *p*‐values below 0.05 were considered significant.

## 3 Results

### 3.1 FAM30A Was Down‐Regulated in CRC

**FIGURE 1:**
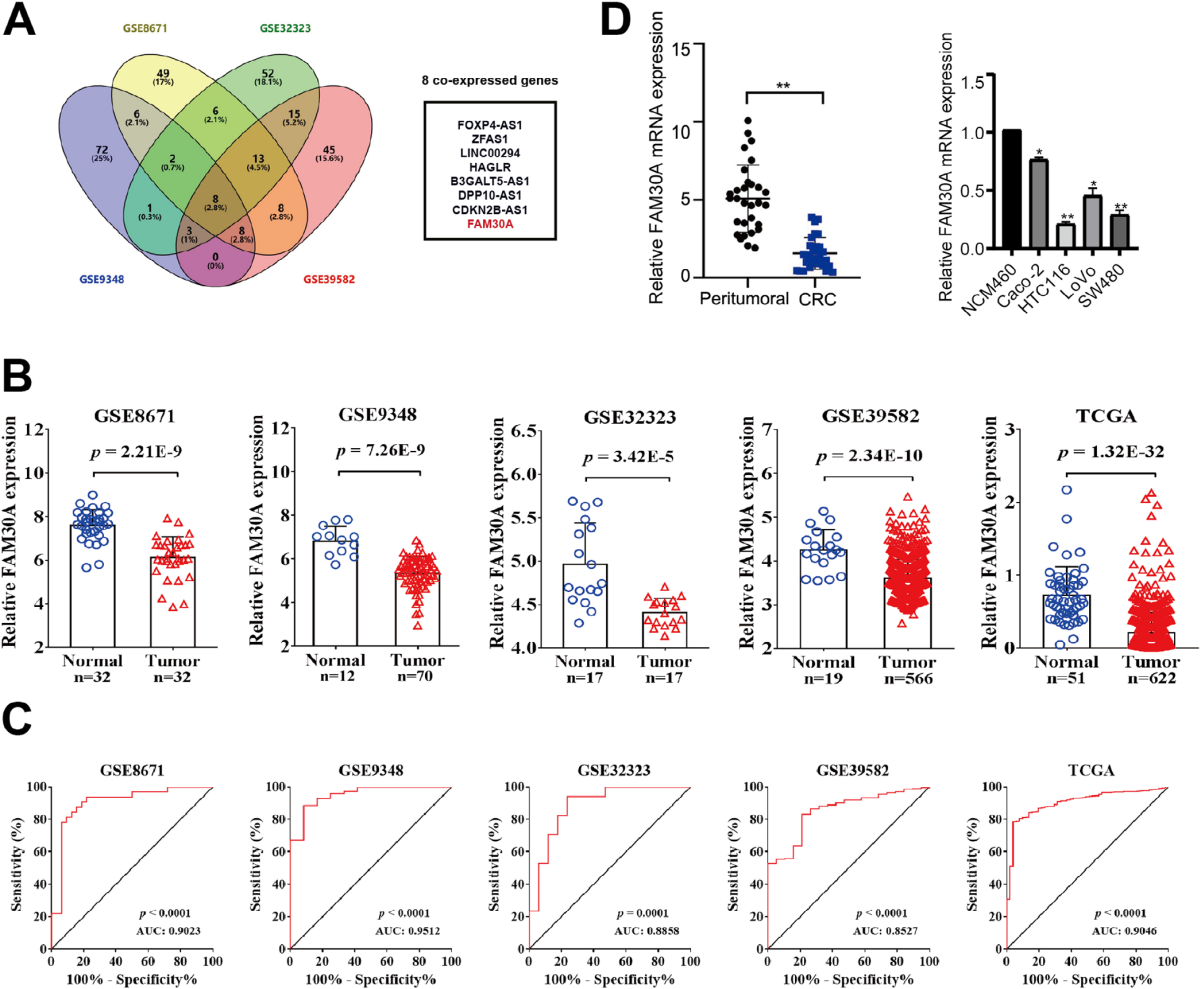
Expression analysis of FAM30A. (A) Venn diagram for common DEGs from four GEO datasets. (B) The expression level of FAM30A in four GEO datasets and TCGA. (C) ROC curves for the expression of FAM30A in four GEO datasets and TCGA. (D) The expression level of FAM30A in clinical samples and in CRC cell lines.

Microarray data were downloaded from four GEO datasets. DEGs (CRC vs. compared surrounding tissues) in these datasets were identified. The top 100 genes with the most significant differential expression in four GEO dataset were displayed in the form of the heat map (Figures S1–S4). Eight genes, FOXP4‐AS1, ZFAS1, LINC00294, HAGLR, B3GALT5‐AS1, DPP10‐AS1, CDKN2B‐AS1, and FAM30A were found to be differentially expressed in CRC in all four studies (Figure 1A). Among these eight genes, only LINC00294 and FAM30A have not been reported in the literatures about CRC, and FAM30A was under‐expressed in CRC. Therefore, FAM30A was chosen as the research object. The expression of FAM30A in four GEO datasets and TCGA was shown in Figure 1B. The results showed that FAM30A was down‐regulated in all datasets. We also plotted receiver operating characteristic (ROC) curves to verify the above conclusions. As shown in Figure 1C, all ROC curves distributed on the upper left corner of the axis and AUC values all closed to 1, indicating that our analysis of FAM30A expression in different databases is suitable and objective. FAM30A expression was also detected in clinical samples and CRC cell lines. As shown in Figure 1D, compared with peritumoral samples or normal colonic epithelial cell lines, FAM30A detected in CRC samples and CRC cell lines both showed lower expression.

### 3.2 FAM30A Regulated Cellular Immune Infiltration and Cancer‐Related Pathways

**FIGURE 2:**
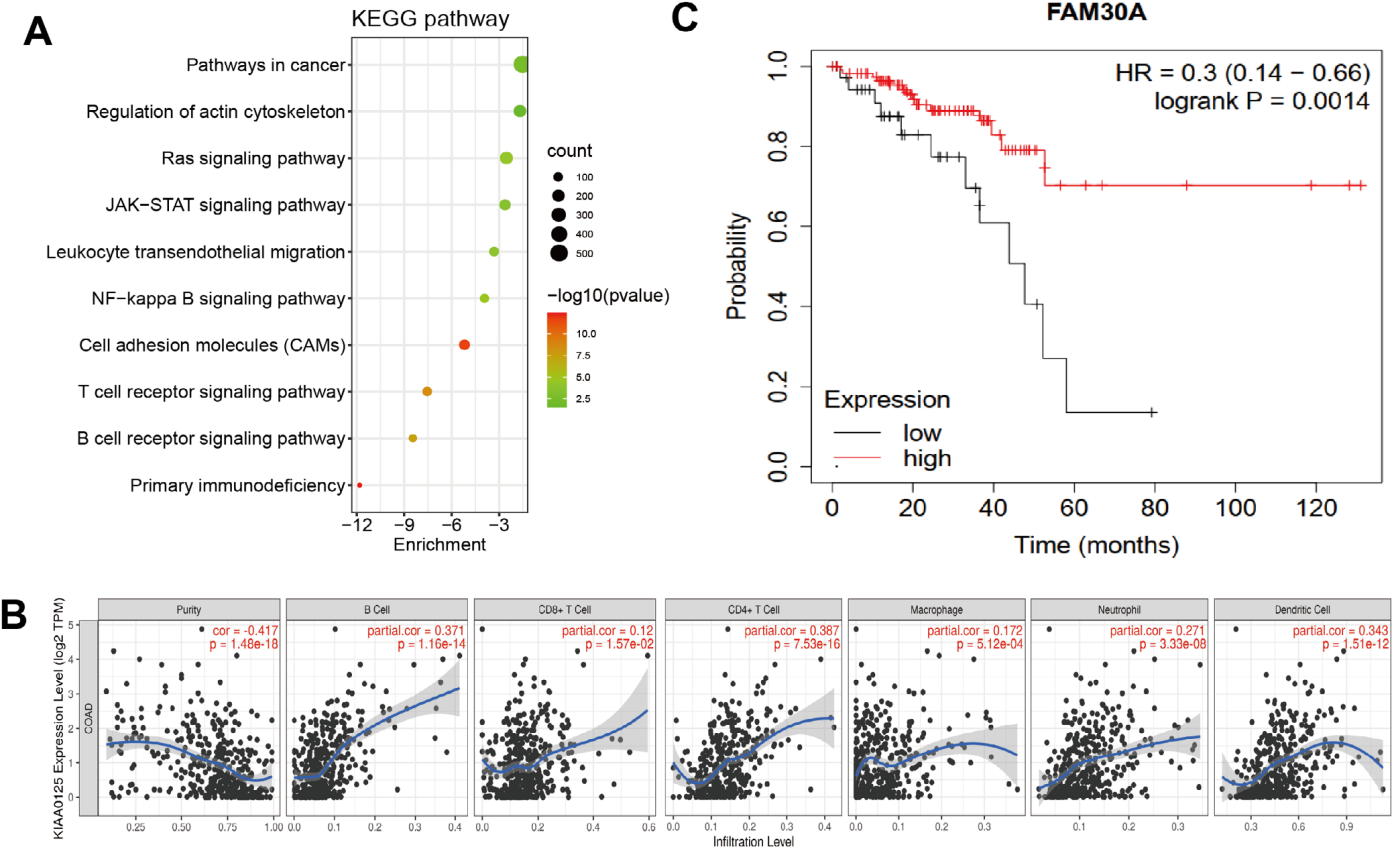
FAM30A regulated cellular immune infiltration and cancer‐related pathways. (A) Enrichment of KEGG pathways for the genes co‐expressed with FAM30A. (B) ESTIMATE algorithm about the expression level of FAM30A and cellular immune infiltration. (C) Correlation between FAM30A's expression level and the survival rate of patients.

Five hundred and thirty‐three common genes co‐expressed with FAM30A were identified from colon (COAD) and rectal (READ) adenocarcinomas in TCGA by using GEPIA. Then, KEGG enrichment analysis was conducted on these 533 genes. The top 10 significantly enriched pathways, including B‐cell receptor signalling pathway, T‐cell receptor signalling pathway, JAK–STAT signalling pathway, and NF‐κB signalling pathway are displayed in Figure 2A. Then, the correlation between the expression of FAM30A and immune cell infiltration, including B cells, CD4^+^ T cells, CD8^+^ T cells, dendritic cells, macrophages, and neutrophil cells was evaluated by using ESTIMATE algorithm (Figure 2B). The results showed that the expression of FAM30A had a strong positive correlation with B cells (*p* = 1.16e‐14), CD4^+^ T cells (*p* = 7.53e‐16), and dendritic cells (*p* = 1.51e‐12), indicating that FAM30A played an important role of cellular immune infiltration. Survival analysis was performed to detect whether FAM30A expression was related to overall survival, and the results showed that high FAM30A expression was positively correlated with better prognosis in CRC patients (Figure 2C).

### 3.3 Overexpressed FAM30A Restrain Colon Cancer Cells to Proliferate and Migrate

**FIGURE 3:**
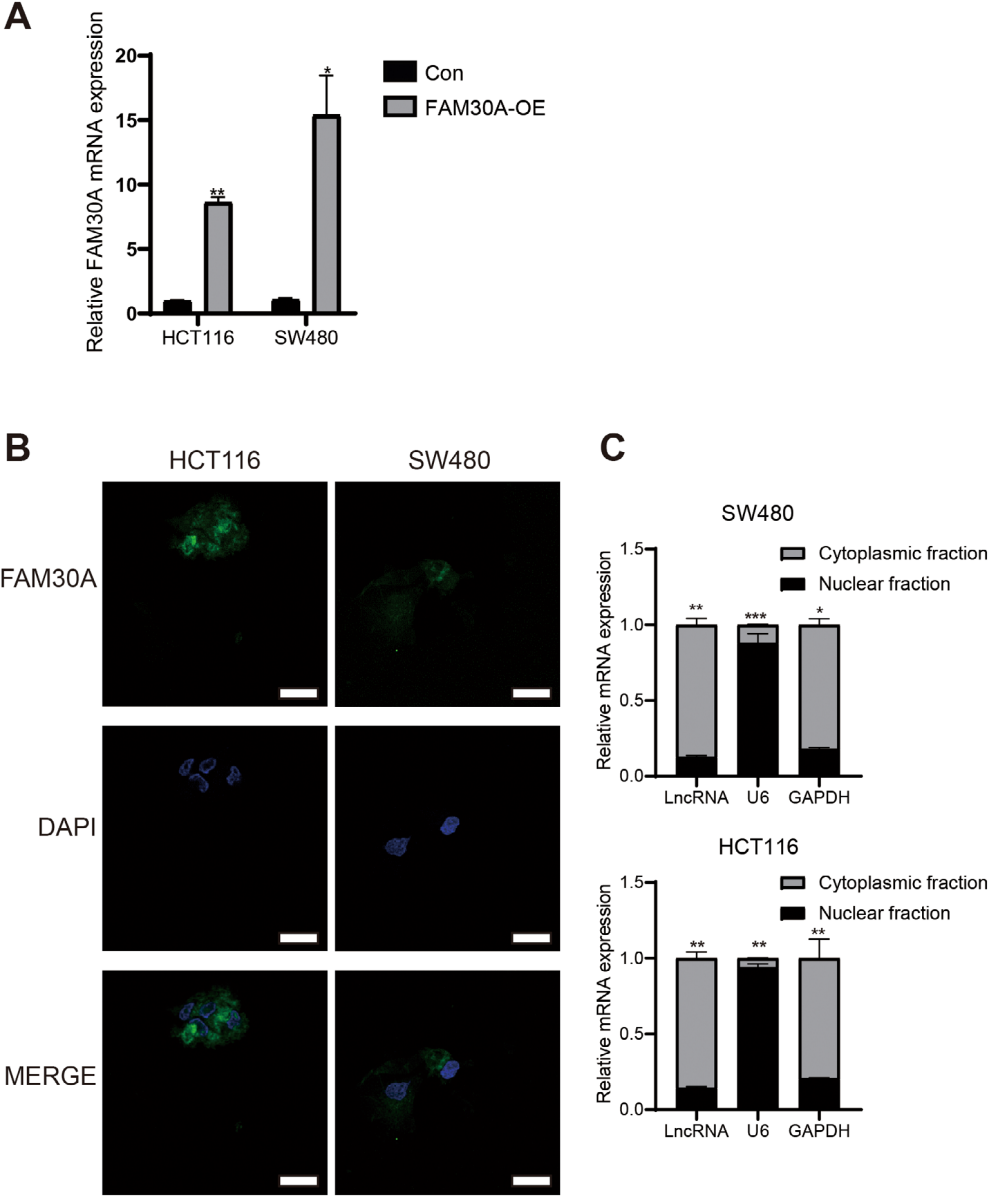
FAM30A expression after transfection and location of FAM30A in cytoplasm. (A) FAM30A's expression level was increased after cell transfection in HCT116 and SW480 cell lines. “con” indicated that cells were transfected with the empty vector (pcDNA3.1) and “FAM30A‐OE” indicated that cells were transfected with FAM30A overexpression vector (pcDNA 3.1 FAM30A). (B) Intracellular localisation of FAM30A. Scale bar = 20 μm. (C) The relative expression of FAM30A in cytoplasmic and nuclear fraction, separately. Each bar represents a gene, with the black and grey portions of each bar representing the mRNA levels of the gene in the nuclear and cytoplasmic fraction, respectively. In the HCT116 and SW480 cell lines, the statistical differences in the ratio of each gene between the cytoplasm and nucleus are as follows. SW480, LncRNA (cytoplasmic fraction vs. nuclear fraction): ****p < 0.0001; U6 (cytoplasmic fraction vs. nuclear fraction): ****p < 0.0001; GAPDH (cytoplasmic fraction vs. nuclear fraction): ****p < 0.0001; HCT116, LncRNA (cytoplasmic fraction vs. nuclear fraction): ****p < 0.0001; U6 (cytoplasmic fraction vs. nuclear fraction): ****p < 0.0001; GAPDH (cytoplasmic fraction vs. nuclear fraction): **p = 0.0013.

**FIGURE 4:**
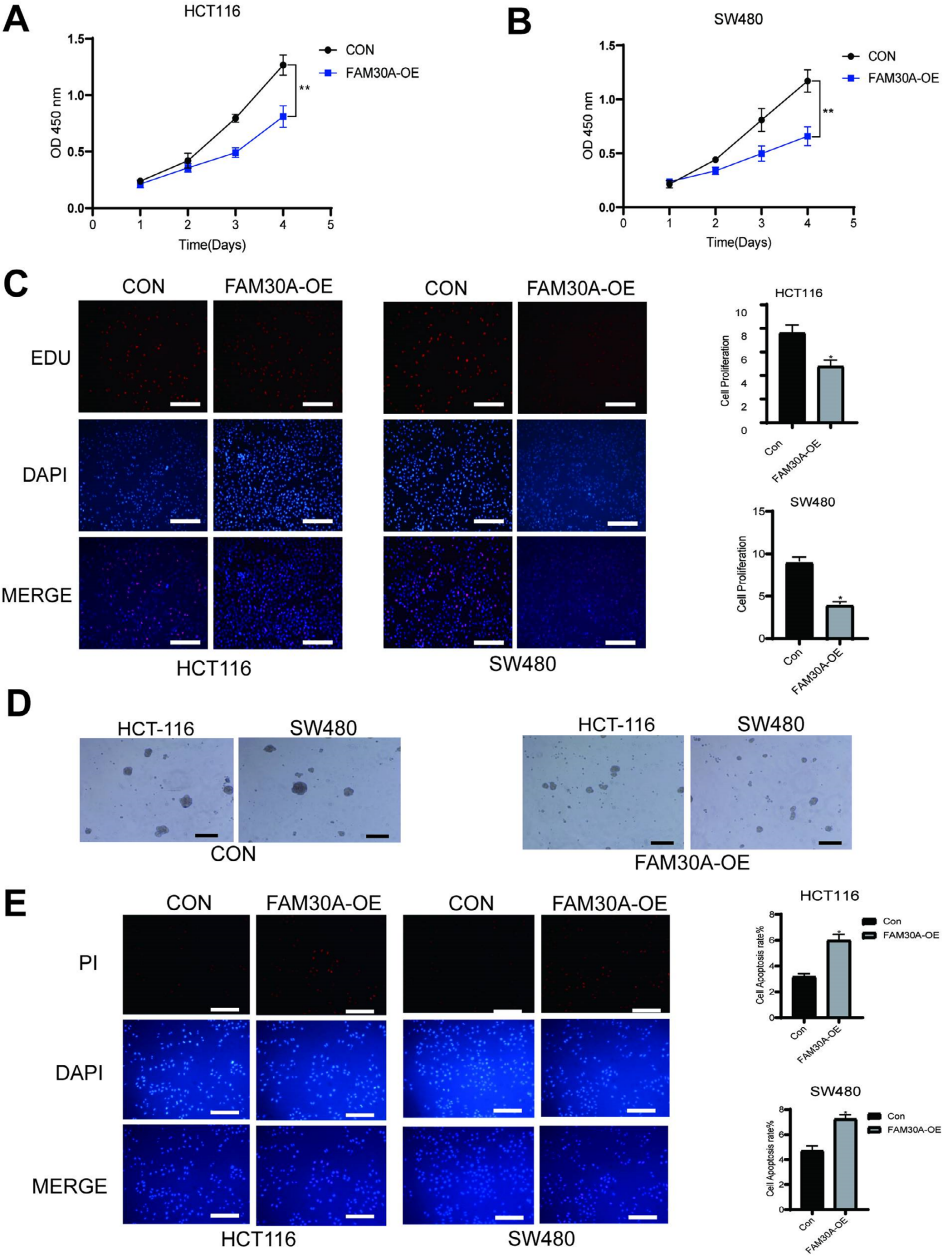
FAM30A inhibited colon cancer cell proliferation and viability. (A) Overexpressing FAM30A decreased the proliferation of HCT116 cell line. (B) Overexpressing FAM30A decreased the proliferation of SW480 cell line. **p < 0.01. (C) Overexpressing FAM30A decreased cell viability of HCT116 cell line and SW480 cell line. Scale bar = 20 μm. *p < 0.05. (D) FAM30A decreased the cell spheroidisation ability of HCT‐116 and SW480 cell lines. Scale bar = 100 μm. (E) Overexpressed FAM30A promoted cell apoptosis of HCT116 cell line. Scale bar = 20 μm. *p < 0.05. “con” indicated that cells were transfected with the empty vector (pcDNA3.1) and “FAM30A‐OE” indicated that cells were transfected with FAM30A overexpression vector (pcDNA 3.1 FAM30A).

**FIGURE 5:**
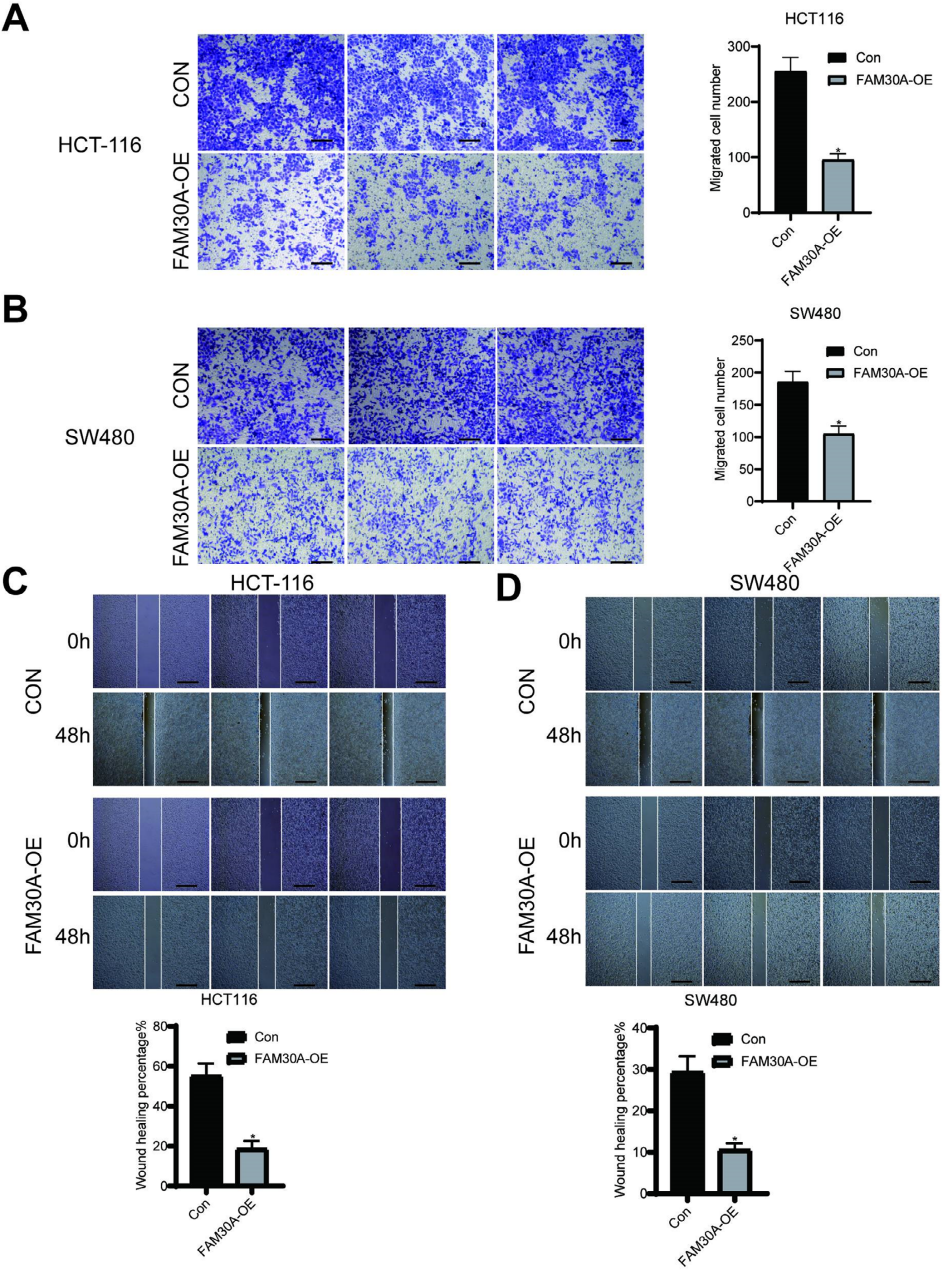
Overexpressing FAM30A decreased colon cancer cell invasion ability and metastasis ability. (A) Overexpressing FAM30A inhibited the invasion ability of HCT‐116 cell line. Scale bar = 20 μm. (B) Overexpressing FAM30A inhibited the invasion ability of SW480 cell line. Scale bar = 20 μm. *p < 0.05. (C) Overexpressing FAM30A inhibited metastasis ability of HCT‐116 cell line. Scale bar = 20 μm. (D) Overexpressing FAM30A inhibited metastasis ability of SW480 cell line. Scale bar = 40 μm. *p < 0.05. “con” indicated that cells were transfected with the empty vector (pcDNA3.1) and “FAM30A‐OE” indicated that cells were transfected with FAM30A overexpression vector (pcDNA 3.1 FAM30A).

Above analyses and experiments showed that FAM30A expression was reduced in CRC patients and positively correlated with favourable prognosis. Consequently, we delved further into investigating the associated mechanisms. FAM30A expression was significantly increased in two CRC cell lines (HCT116 and SW480) after transfection (Figure 3A). Considering the function of lncRNAs was closely related to their subcellular location, the sub‐cellular distribution of FAM30A in CRC cells was analysed. The results of nuclear and cytoplasmic separation assay showed that FAM30A located in cytoplasmic fraction in both CRC cell lines (Figure 3B,C), suggesting that FAM30A might play an important role in the cytoplasm. As shown in Figure 4A–C, after overexpressing FAM30A, the proliferation of CRC cells was significantly suppressed (*p* < 0.01) and the viability of CRC cells was weakened. Overexpressing FAM30A also weakened the cell spheroidisation ability of colon cancer cells (Figure 4D). To confirm whether FAM30A could affect apoptosis in HCT116 and SW480 cells, a TUNEL assay was performed. As shown in Figure 4E, the number of TUNEL‐positive cells was increased in the FAM30A overexpressed group compared with control group. Next, the effect of FAM30A on the capacity of metastasis and invasion of CRC cells was detected. From the results of transwell assay and wound‐healing assay, it was found the capacity of metastasis and invasion of CRC cells was significantly weakened (Figure 5).

### 3.4 FAM30A Blocked the JAK–STAT Signalling

**FIGURE 6:**
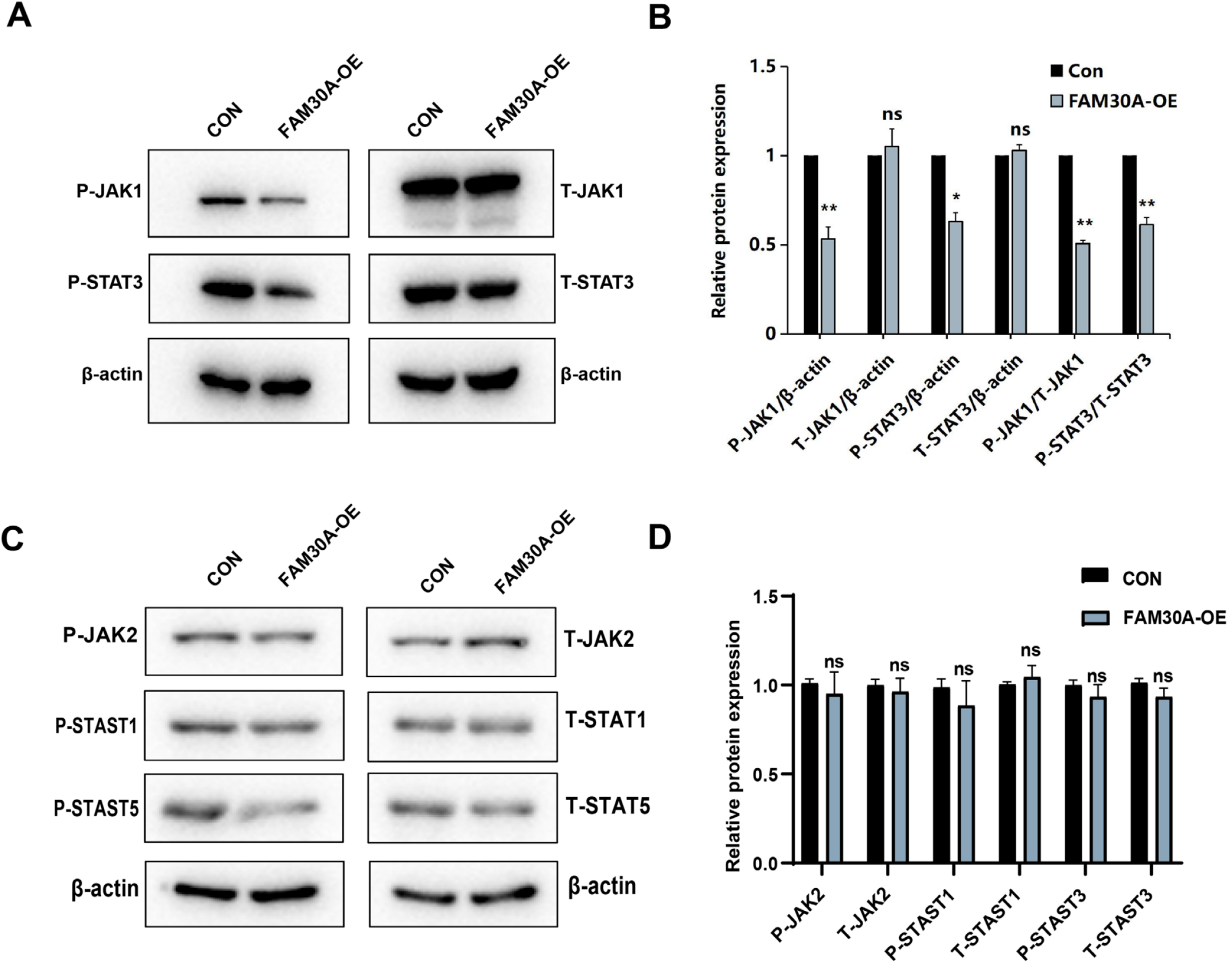
Impact of FAM30A Overexpression on JAK1/STAT3 Signalling. (A) Western blot analysis comparing phosphorylated and total JAK1/STAT3, with β‐actin as a loading control. (B) Relative quantification of P‐JAK1, P‐STAT3, T‐JAK1, and T‐STAT3 levels normalised to β‐actin, with the ratios of P‐JAK1/T‐JAK1 and P‐STAT3/T‐STAT3. (C) Western blot analysis comparing phosphorylated and total JAK2/STAT1/STAT5, with β‐actin as a loading control. (D) Relative quantification of P‐JAK2, P‐STAT1, P‐STAT5, T‐JAK2, T‐STAT1, and T‐STAT5 levels normalised to β‐actin. *p < 0.05, **p < 0.01. “con” indicated that cells were transfected with the empty vector (pcDNA3.1) and “FAM30A‐OE” indicated that cells were transfected with FAM30A overexpression vector (pcDNA 3.1 FAM30A).

Previous studies have shown that JAK–STAT signalling pathway plays an important role in the proliferation and migration of CRC [42, 43]. As overexpressing FAM30A significantly weakened the viability of CRC cells, the expression levels of protein involved in JAK–STAT signalling pathway therefore examined to explore the downstream molecular mechanism of FAM30A in CRC. Phosphorylation levels of JAK1 and STAT3 were significantly decreased after FAM30A overexpression, indicating that the activity of JAK1–STAT3 signalling was decreased after FAM30A overexpression (Figure 6A,B).

To further validate the specific role of FAM30A in the JAK1–STAT3 signalling pathway, we extended the correlation experiments to examine the phosphorylation and total protein levels of JAK2, STAT5, and STAT1. As shown in Figure 6C,D, FAM30A overexpression had no significant effect on the phosphorylation and total protein levels of JAK2, STAT5, and STAT1. This supports that FAM30A mainly specifically regulates the activity of JAK1 and STAT3 without significantly affecting the activity of other JAK/STAT members.

## 4 Discussion

### 4.1 Main Interpretation

FAM30A is an antisense oriented transcript associated with a gene fragment at the IgH locus, with the popularisation of high‐throughput sequencing technology, its function on genome and transcriptome have gradually been explored [44]. The role of FAM30A in cancer is only reported in laryngeal squamous cell carcinoma [45], gastric cancer [46] and chronic lymphocytic leukaemia [47], and in‐depth studies are lacking. In this study, we first report the role of FAM30A in the process of CRC. The analysed of bioinformatic found that FAM30A showed low expression in CRC and high FAM30A expression was correlated with good prognosis. Low expression of FAM30A was also validated in clinical samples and four CRC cell lines. Further, we preliminarily explored the role of FAM30A in the occurrence and development of CRC through in vitro experiments. Overexpression of FAM30A could inhibit the proliferation, invasion and metastasis of CRC, and promote CRC cell apoptosis. Moreover, overexpressing FAM30A can block JAK–STAT signalling pathway. FAM30A may regulate the proliferation, invasion and metastasis of CRC through JAK–STAT signalling. It is worth noting that the function of lncRNAs is closely related to their subcellular location. LncRNAs operating within the nucleus versus the cytoplasm can exhibit distinct molecular mechanisms [48]. Highlighting this separation enables a more precise understanding of their roles and potentially facilitates the discovery of different functional pathways within the cell. In this study, we also analysed the sub‐cellular distribution of FAM30A in CRC cells, and found that FAM30A was mainly distributed in the cytoplasm, but also distributed in the nucleus. This suggests that FAM30A may play an important role in the cytoplasm. However, the localisation‐dependent mechanism of FAM30A in CRC remains to be further explored.

As a cDNA, FAM30A was expressed in the spleen, KG‐1 cell line, and peripheral blood [48]. In the present study, KEGG analysis was performed on the co‐expressed genes of FAM30A and several signalling pathways including B‐cell receptor signalling pathway, T‐cell receptor signalling pathway, NF‐кB signalling pathway, JAK–STAT signalling pathway, and Ras signalling pathway was found, which were consistent with the analysis of tumour immune microenvironment of CRC. Immune cells infiltration in tumour closely associated with prognosis of tumour. High FAM30A expression was positively correlated with better prognosis in CRC patients, which may be achieved by higher infiltration of B cells, CD4^+^ T cells, and dendritic cells in CRC and benefited by B‐cell receptor signalling pathway, T‐cell receptor signalling pathway. These findings provided valuable insights into potential future directions. We will further investigate the underlying mechanisms by which FAM30A expression influences the infiltration of immune cells in CRC and how they contribute to anti‐tumour immune responses. A series of experiments will be carried out, such as overexpressing FAM30A in different immune cells, animal experiments, and RNA‐Seq. This can help clarify the molecular pathways involved.

Consistent with our conclusion that high FAM30A expression was correlated with good prognosis, some studies have pointed out that FAM30A may be a potential biomarker. Akrami et al. [49] pointed out that FAM30A plays a crucial role in the process of pancreatic ductal adenocarcinoma. Wu et al. [50] found that FAM30A is an immune‐related prognostic factor in lung adenocarcinoma. Kaplan–Meier analysis and ROC analysis showed that eight‐lncRNA‐based models can accurately predict the prognosis of LUAD patients. Fang et al. [51] found that M2 macrophage metabolism is closely related to lung cancer, and FAM30A participates in the M2 macrophage intracellular metabolic pathway, which shows good prognosis prediction ability.

The research on CRC targeted therapy strategies mainly focuses on monoclonal antibodies and small molecule inhibitors, while the research of drug delivery mainly focuses on nanoparticle‐based delivery and liposomes [52, 53]. For instance, Chen et al. [54] found that Vitexin induces M1 polarisation of macrophages in the tumour microenvironment via the VDR/PBLD signalling pathway, mitigating the progression of CRC in mice. Besides, the research by Xu et al. revealed that targeting tsRNA‐GlyGCC in combination with 5‐FU may provide a promising nanotherapeutic strategy for the treatment of 5‐FU‐resistance CRC [55].

LncRNA has been implicated in the regulation of key signalling pathways involved in CRC progression [56]. Based on the results in this research, understanding FAM30A specific role in CRC can help inhibit the tumour progress and enhance the clinical applicability. The subsequent studies could focus on the combination therapies: Combining FAM30A‐targeted therapies with existing treatments (such as chemotherapy or immunotherapy) may enhance overall treatment efficacy and overcome resistance mechanisms commonly seen in CRC.

### 4.2 Limitations

However, there is currently little research on taking FAM30A as a therapeutic target for CRC. Although we have validated that overexpression of FAM30A could inhibit the proliferation, invasion, and metastasis of CRC, and promote CRC cell apoptosis, much more experiments needed to explore whether FAM30A could be a novel therapeutic target and when and at what stage of colorectal cancer cases will it be administered and with what delivery system? Combining FAM30A and immune checkpoint inhibitor may perform a new insight. Besides, it is crucial to keep a healthy lifestyle, regular checkups, screenings and vaccines for cancer prevention. To determine the potential of FAM30A as a tool for CRC prevention, further experiments are also needed to validate its efficacy.

In the future, we will expand the sample size to evaluate the expression levels of FAM30A in CRC patients and normal tissues. Furthermore, we will utilise gene knockout and overexpression experiments to investigate its relationship with CRC development. Building upon the existing research, we will establish additional animal models to simulate CRC progression and assess the therapeutic effects of FAM30A. Subsequently, we will conduct a detailed study of the biological changes in vivo following FAM30A treatment, such as immune cell infiltration, and perform toxicity, pharmacokinetic, and pharmacodynamic studies.

### 4.3 Conclusion

LncRNAs are crucial in CRC progression, including proliferation, migration, and invasion and have gradually becoming potential therapeutic targets. Our findings suggest that FAM30A, a newly discovered lncRNA, plays a crucial role in the progression of CRC. FAM30A is down‐regulated in CRC, and its low expression predicts poor prognosis. Overexpression of FAM30A can inhibit CRC cell invasion and metastasis and promote cell apoptosis. Our results provide a better understanding of the role of FAM30A in CRC progression and a potential therapeutic target and prognostic predictor against CRC.

## Author Contributions

**Jin Liu:** supervision (equal), writing – original draft (equal). **Shuangyin Han:** validation (equal). **Yuanbo Cui:** validation (equal), visualization (equal). **Qiuyan Zhao:** investigation (equal). **Yunfei Wang:** methodology (equal), writing – original draft (equal). **Tian Li:** writing – review and editing (equal). **Xiuling Li:** conceptualization (equal).

## Ethics Statement

All aspects of this study have been approved by the Research Ethics Committee of Henan Provincial People's Hospital and the approval number was [2020]‐03.

## Consent

Informed consent was obtained from patients.

## Conflicts of Interest

The authors declare no conflicts of interest.

## Supporting information


**Figures S1–S4.** The heat map of top 100 genes with the most significant differential expression in four GEO dataset.

**Table S1.** Detailed information of each dataset.

**Table S2.** Specific information about all patients.

## Data Availability

The datasets GSE9348, GSE32323, GSE8671, and GSE39582 can be found in GEO. The data generated in the present study are included in the figures and/or tables of this article. Other data are available on https://www.jianguoyun.com/p/DR2mh44QuaiFChiJ‐9UFIAA.
