# Supplementary figures and images for: LncRNA FAM30A Suppresses Proliferation and Metastasis of Colorectal Carcinoma by Blocking the JAK–STAT Signalling
Source: J Cell Mol Med. 2025 Feb 19;29(4):e70421. doi: 10.1111/jcmm.70421 (PMC11839745; doi:10.1111/jcmm.70421)

Supplementary Figures  
Figure S1

GSE8671

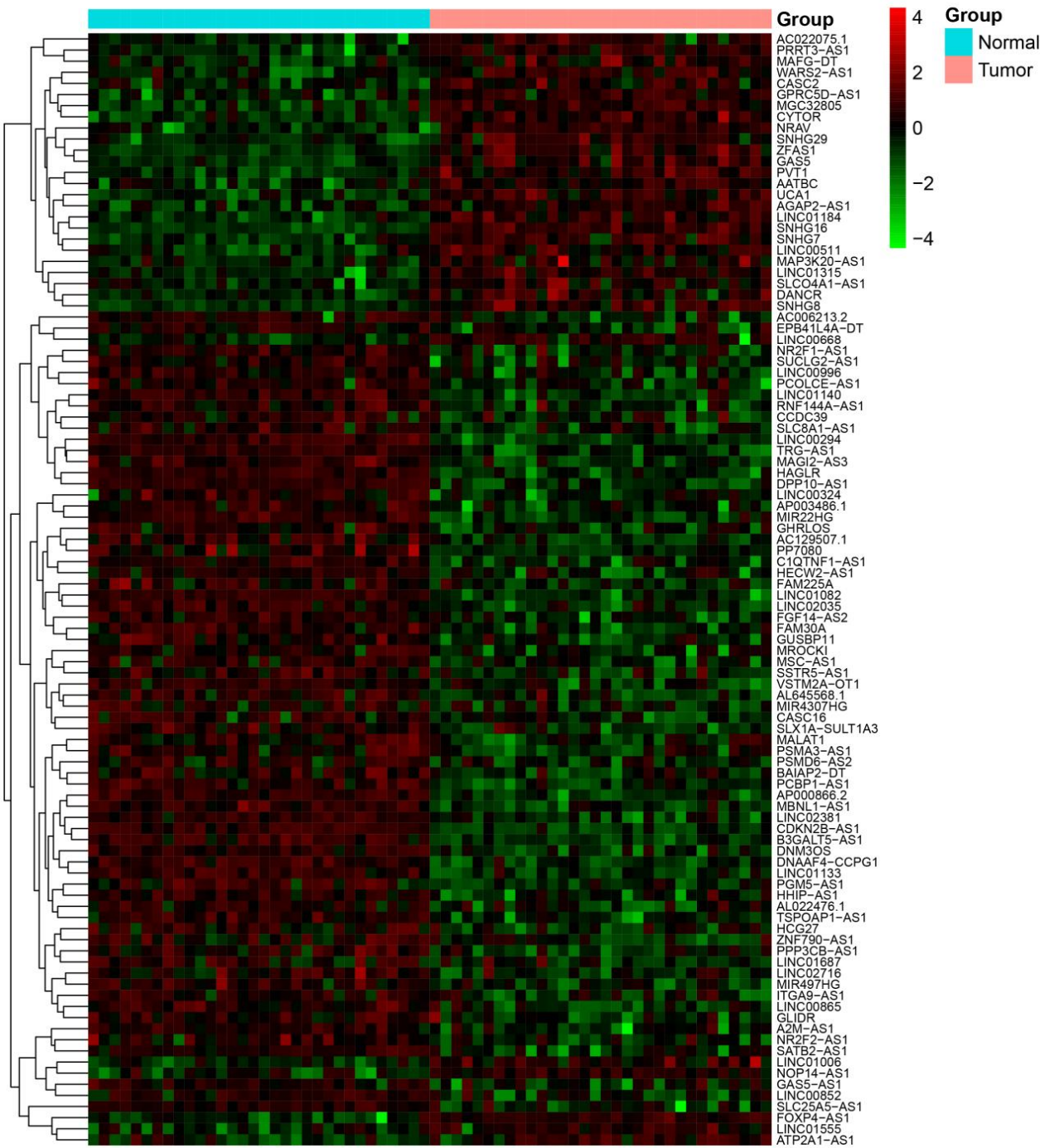

Figure S2

GSE9348

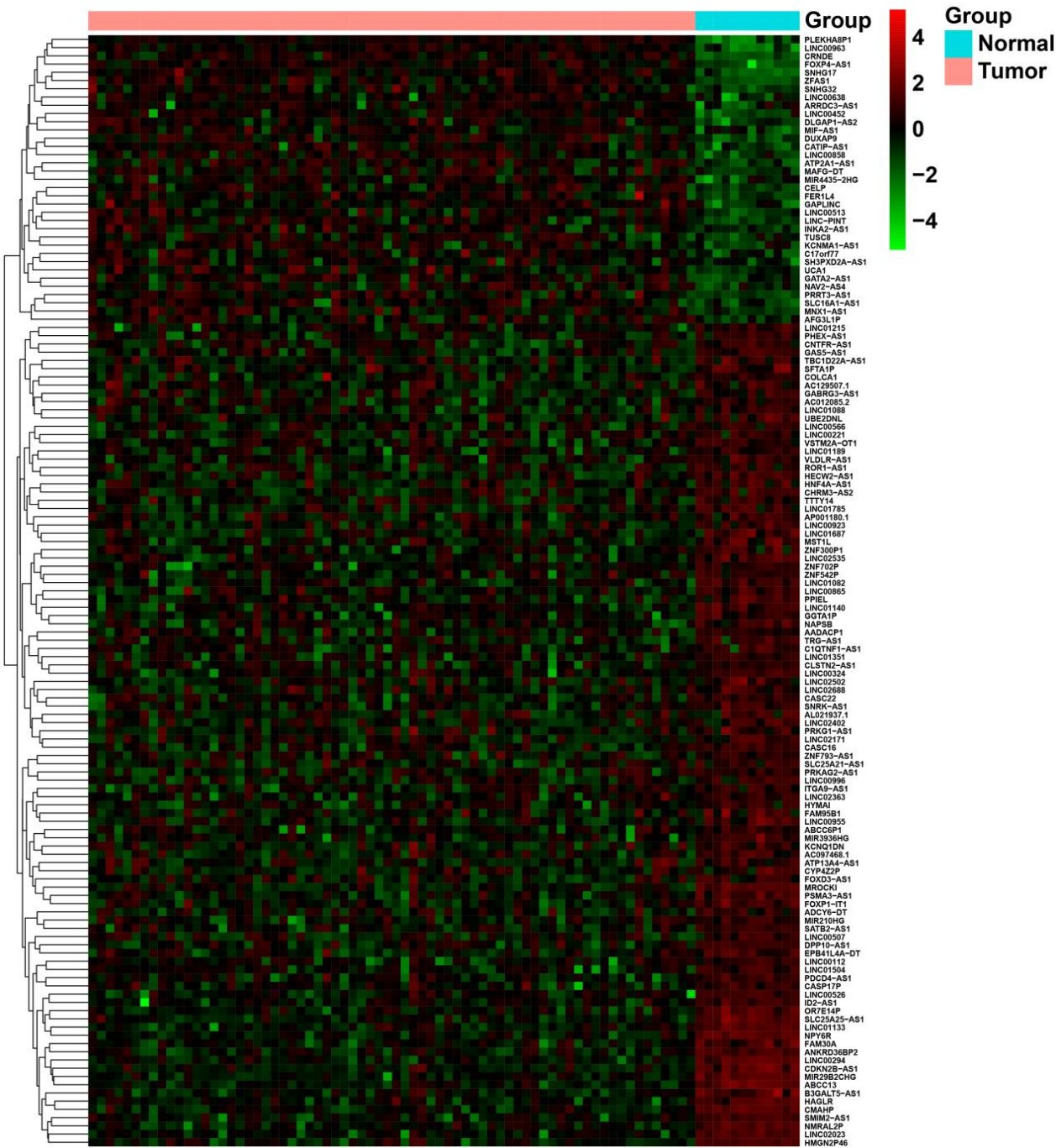

Figure S3

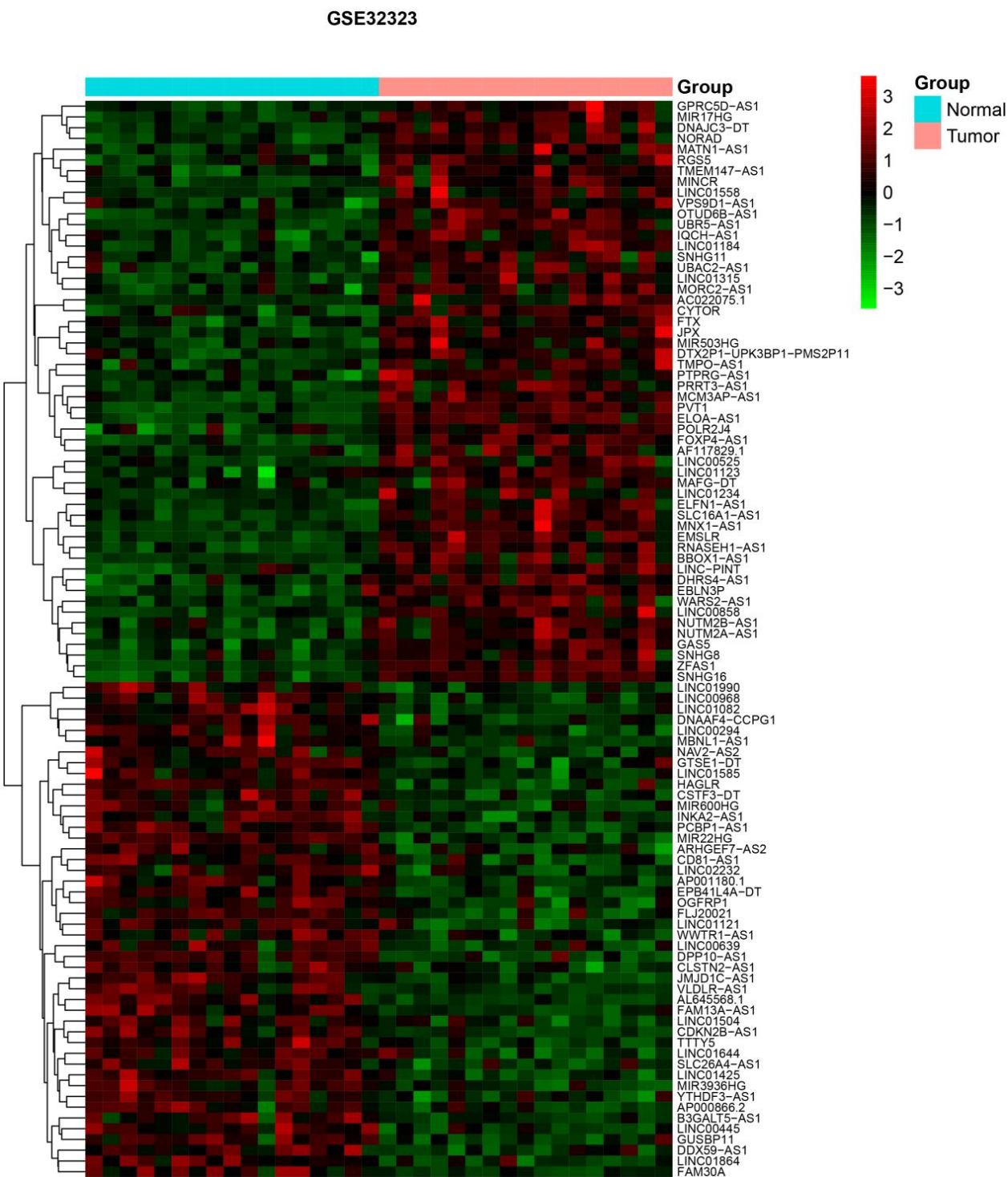

Figure S4

GSE39582

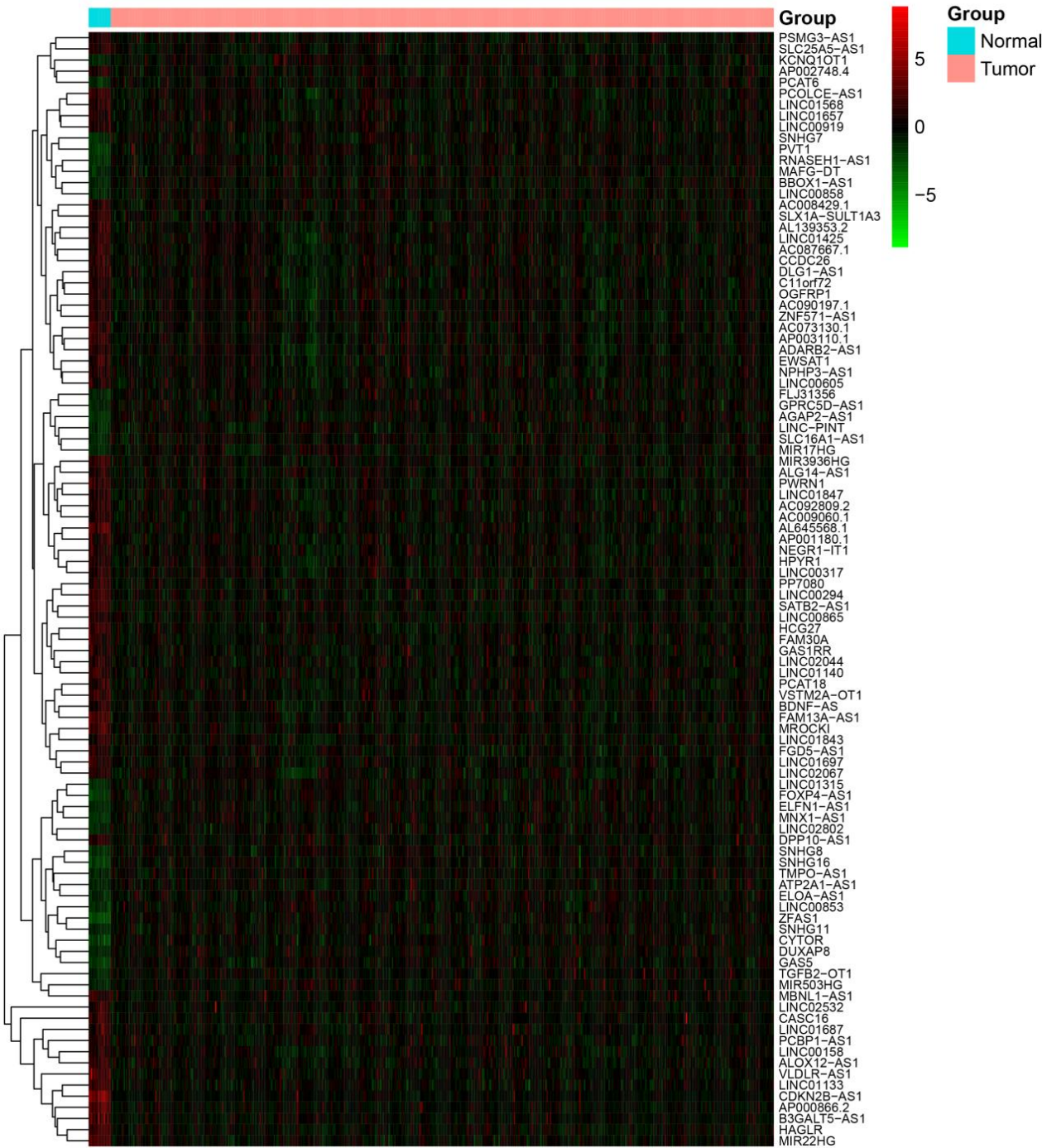

Supplement: Supplementary file 1 — Figures S1–S4. The heat map of top 100 genes with the most significant differential expression in four GEO dataset. [file JCMM-29-e70421-s002.pdf]
