# Supplementary material for: LncRNA FAM30A Suppresses Proliferation and Metastasis of Colorectal Carcinoma by Blocking the JAK–STAT Signalling
Source: J Cell Mol Med. 2025 Feb 19;29(4):e70421. doi: 10.1111/jcmm.70421 (PMC11839745; doi:10.1111/jcmm.70421)
Supplement: Supplementary file 2 — Table S1. Detailed information of each dataset. [file JCMM-29-e70421-s001.docx]

Supplementary table 1. Detailed information of each dataset

| **Dataset** | **Number of samples** | **Inclusion conditions** | **Exclusion conditions** |
| --- | --- | --- | --- |
| GSE9348 | 82 (70 disease samples, 12 control sample) | Aged 50 years or more; Tumors classified as early stage (I/II);  Microsatellite-stable; Left-sided tumors. | High microsatellite instability;  Tumors with colonic perforation or with the resection margins. |
| GSE32323 | 17 pairs (17normal samples, 17 tumor samples) | Samples from 40 patients who underwent surgical treatment for CRC between 2005 and 2007 at Tokyo Medical and Dental University Hospital were included. |  |
| GSE8671 | 28 pairs (28 normal samples, 28 tumor samples) | Type 0-Ip (6); Maximum diameter of 1 to 4 cm; Absence of surface ulceration;  Histologic diagnosis of adenoma; Absence of microsatellite instability at BAT26. | Patients with documented familial polyposis; with>15 adenomatous polyps;  Currently treated with nonsteroidal anti-inflammatory drugs (including aspirin). |
| GSE39582 | 566 (443 discovery samples, 123 validation samples, include 19 adjacent non-tumor tissue ) | Stage I to IV CC. | Received preoperative chemotherapy and/or radiation therapy and those with primary rectal cancer were exclusion. |
