# Supplementary material for: LncRNA FAM30A Suppresses Proliferation and Metastasis of Colorectal Carcinoma by Blocking the JAK–STAT Signalling
Source: J Cell Mol Med. 2025 Feb 19;29(4):e70421. doi: 10.1111/jcmm.70421 (PMC11839745; doi:10.1111/jcmm.70421)
Supplement: Supplementary file 3 — Table S2. Specific information about all patients. [file JCMM-29-e70421-s003.docx]

**Supplementary table 2: Specific information about all patients**

| Patients' Number | Gender | Age (years old) | Pathological staging | Chemotherapy (yes or no) |
| --- | --- | --- | --- | --- |
| 1 | male | 58 | T4N2bM0 | no |
| 2 | male | 52 | T3N0M0 | no |
| 3 | male | 34 | T4N3M0 | no |
| 4 | male | 52 | T4N0M0 | no |
| 5 | male | 52 | T3N0M0 | no |
| 6 | male | 57 | T3N1M0 | no |
| 7 | male | 62 | T3N1M0 | no |
| 8 | female | 55 | T3N0M0 | no |
| 9 | female | 72 | T1N0M0 | no |
| 10 | female | 31 | T3N0M0 | no |
| 11 | female | 53 | T3N1M0 | no |
| 12 | female | 68 | T3N0M0 | no |
| 13 | female | 65 | T3N1M0 | no |
| 14 | male | 55 | T3N1M0 | no |
| 15 | female | 70 | T2N0M0 | no |
| 16 | male | 51 | T3N1M0 | no |
| 17 | female | 76 | T4N2M0 | no |
| 18 | male | 59 | T3N2M0 | no |
| 19 | male | 62 | T3N2MO | no |
| 20 | male | 68 | T3N1M0 | no |
| 21 | male | 61 | T3N0M0 | no |
| 22 | female | 34 | T3N0M0 | no |
| 23 | female | 65 | T3N1M0 | no |
| 24 | female | 55 | T3N0M0 | no |
| 25 | female | 74 | T3N1M0 | no |
| 26 | male | 55 | T3N0M0 | no |
| 27 | female | 41 | T4aN0M0 | no |
| 28 | male | 55 | T3N1M0 | no |
| 29 | female | 32 | T2N0M0 | no |
| 30 | female | 44 | T3N1M0 | no |
